# The Influence of Video Game Training with and without Subpatelar Bandage in Mobility and Gait Speed on Elderly Female Fallers

**DOI:** 10.1155/2018/9415093

**Published:** 2018-03-26

**Authors:** Isabela Feitosa de Carvalho, Gianluca Loyolla Montanari Leme, Marcos Eduardo Scheicher

**Affiliations:** ^1^Human Development and Technologies, UNESP, Rio Claro, SP, Brazil; ^2^Physical Therapy, UNESP, Marília, SP, Brazil

## Abstract

**Objectives:**

The aim of the study was to investigate the effect of balance training with Nintendo Wii technology, with and without the use of additional sensory information (subpatellar bandage), in the functional mobility and gait speed of elderly female fallers.

**Methods:**

Twenty elderly women were divided into two groups: group I: trained with the use of the Nintendo Wii; group II: trained using the Nintendo Wii and the addition of sensory information (subpatellar bandage). The functional mobility was assessed with the *Timed up and Go* test (TUG) and gait speed with the 10 m test. The tests were carried out with and without the use of the subpatellar bandage. The training was carried out within sessions of 30 minutes, twice a week, using three different games (*Penguin Slide*, *Table Tilt*, and *Tightrope*).

**Results:**

There was an increase in the gait speed and a decrease in the TUG time in both groups, independently of the sensory condition used (*p* < 0.05). In the short term, the subpatellar bandage improved the TUG time (*p* < 0.05) and the gait speed (*p* < 0.01).

**Conclusion:**

The training for postural balance with virtual reality was effective for improving functional mobility and gait speed of elderly female fallers. The subpatellar bandage did not maximize the effect of training.

## 1. Introduction

The use of video games for the training of postural balance is increasing in research related to the elderly population, due to the favorable results on the improvement of posture control, as well as a decrease in the risk of falls [1–3].

Studies in elderly established that many aspects of motor control are affected by aging, causing slower movement and less coordinated [4]. The negative effect of age on balance stability is attributed primarily to sensorimotor dysfunctions [5, 6], muscle weakness [7], and structural changes in brain grey and white matter [8, 9].

For balance maintenance, the postural control system uses mainly information from three sensory systems (visual, vestibular, and somatosensory), which provide information about the relative positions of the body segments and the magnitude of the forces acting on this body, which are not constant [10–12]. Because of this, it is reasonable to suggest that the body orientation is obtained by a relationship between sensory information and motor action. In this case, sensory information influences the performance of the motor actions related to the postural control and, simultaneously, the accomplishment of these motor actions influences the obtaining of sensory information [12].

In this sense, some studies have investigated the effect of somatosensory information on postural control [13–16]. These studies indicate that sensory information can be added with a contact on a touch bar or with a subpatellar bandage, causing a reduction in body oscillation. In these studies, the beneficial effects of the use of additional sensory information were observed in healthy individuals and in individuals with an anterior cruciate ligament injury. In other studies, similar results have been found in the elderly fallers [17–19] using a subpatellar bandage as additional sensory information.

In a recent research paper, Botelhos and Bonfim [20] showed that the effects of a sensorimotor training can be improved with the use of the additional sensory information in healthy individuals.

However, although these studies demonstrate a positive effect of the use of additional sensory information on postural control, there is no evidence that this increasement in sensory input can contribute in a dynamic intervention process, such as in postural balance training with video games in elderly fallers.

Therefore, the objective of this study was to investigate the effect of balance training with Nintendo Wii® technology, with and without the use of additional sensory information (subpatellar bandage), in the functional mobility and gait speed of elderly female fallers.

## 2. Methodology

Twenty elderly women who live in Marilia, Sao Paulo, 60 years old or over, classified as sedentary according to the Brazilian Society of Sports Medicine and the Brazilian Society of Geriatrics and Gerontology [21], with a history of one or more falls in the last 12 months were recruited for the study [22]. All patients were recruited in geriatrics clinics, community centers, primary care assistance, basic health units, and so on.

Only the women who had the ability of independent ambulation without the use of walking aid devices were selected. Their cognitive abilities were evaluated with the Mini-Mental State Examination (MMSE) and the elderly who obtained scores considered normal for their respective years of study were included in the study [23]. The women who presented sequelae of neuromusculoskeletal diseases, arthritis, uncorrected vision problems, and postural hypertension and reported the continuous use of sedatives, antidepressants, and hypnotics were not included in the study.

All participants answered questionnaires regarding their personal data, history of falling (place, number, and sequelae), marital status, and number of medications.

**Figure 1:**
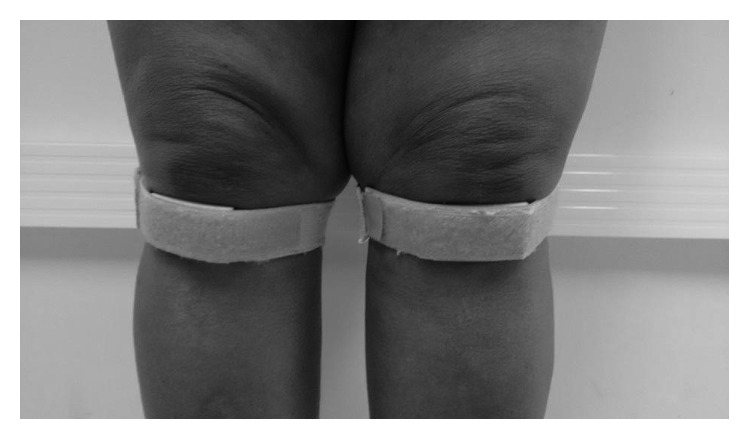
Subpatellar bandage.

The participants were separated into two groups:

1. Training without additional sensory information (untaped condition, control group)
2. Training with additional sensory information (taped condition): subpatellar bandage condition with a pad, a width of 2 cm, Salvape® brand, maintaining contact with the skin bilaterally, in an orthostatic position (Figure 1).

## 3. Assessment Section

### 3.1. Functional Mobility

The mobility was evaluated with the TUG test, before and after training. The test measures the time (in seconds) necessary for a person to rise from a chair with armrests, walk 3 meters at a comfortable walking speed, turn, return to the chair, and sit down [24].

The test was performed twice in the untaped and taped condition, first for familiarization and then for time recording [24]. The TUG test is highly recommended as a means of assessing the risk of falling for the elderly because it identifies the deficit of balance and gait speed. Therefore, lower scores indicate better functional mobility, better posture, and an increased gait speed [25].

### 3.2. Gait Speed

In order to evaluate the gait speed, a simple 10-meter walking test was carried out. The usual gait speed is a simple measure, and there is no need for expensive and sophisticated equipment, but simply the use of a timer and some walking space. In order to eliminate any anomalies, the participants had to begin walking 1.2 meters before the timing and finish 1.2 meters after [26]. A single examiner using a digital stopwatch with a 1/100 of a second reading (Cronobio SW-2018^®^, Pastbio, SP, Brazil) recorded the walking time of all volunteers.

The test was carried out three times in order to eliminate any variables; the shortest durations were used in the data and were analyzed. The participants were told to continue walking to the next point, in their regular speed as soon as they were ready [26, 27].

## 4. Balance Training with Virtual Reality

The postural balance training was carried out within sessions of 30 minutes, twice a week. The exercises were done with commercialized games of *Wii Fit by Nintendo*® in sync with the *Wii Balance Board*®. This platform is wireless and connects to the *Nintendo Wii* console via Bluetooth. It has a rectangular design and includes sensory pressure that detects the center of pressure and movement of the user [28, 29].

Three different games were used for postural balance training: *Penguin Slide*, where the participants had to catch fish while balanced on a piece of ice by shifting their weight from side to side; *Table Tilt*, where the participants had to shift body weight in multiple directions to get balls into a series of holes; and *Tightrope*, where the participants had to cross a tightrope by marching in place and do verticals jumps by bending and extending their knees to avoid dynamic obstacles. The personal profile of the participant was entered into the system so that his or her progress could be tracked and difficulty levels could be adjusted to each person's training program according to his/her progress [28]. All the evaluations were carried out by the same evaluator.

Each game had a duration of 10 minutes [30]. The participants observed the body movements on a multimedia projector, providing greater visual feedback.

**Figure 2:**
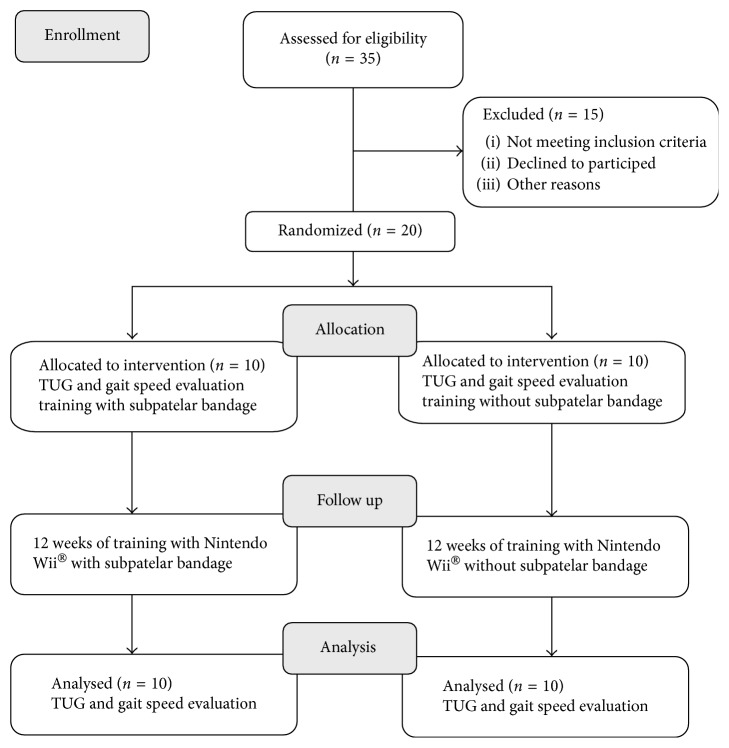
Methodology flowchart.

The intervention went on for 12 weeks, with two sessions per week on different days. The methodology flowchart can be seen in Figure 2. At the beginning and the end of each session, the blood pressure, heart rate, and breathing were checked.

Written informed consent was obtained from all participants before enrollment. The study was approved by the Research Ethics Committee of the Faculty of Philosophy and Sciences–UNESP (process no. 0982/2014) and published in the Brazilian Registry for Clinical Trials (RBR-54jrwd).

## 5. Data Analysis

The normality of the distribution of data was evaluated by the *Shapiro–Wilk* test. A repeated measures ANOVA with Bonferroni posttest (within-subjects) was used to compare the data. All analysis were made using GraphPad Instat software, with *p* < 0.05.

## 6. Results

**Figure 3:**
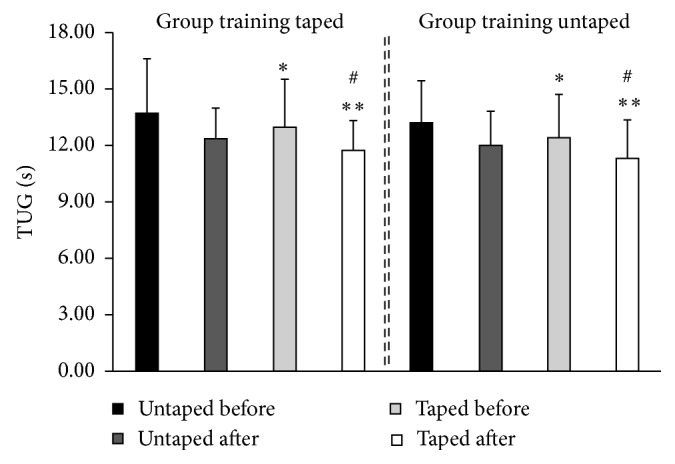
Comparison of TUG test between taped and untaped groups before and after training. ^∗^*p* < 0.05 in comparison to untaped before; ^∗∗^*p* < 0.01 in relation to untaped before; ^#^*p* < 0.05 in comparison to taped before.

The Shapiro–Wilk test showed that the data had a normal distribution.  Table 1 demonstrates the participants' characterizations in terms of age, test scores (MMSE), and their number of falls. Of the sample evaluated, 50% of the women reported that their falls had been frequent the year before. All the participants used medication, and 70% of them used more than one type of medication per day.  Figure 3 represents the measurements of the TUG test before and after training, with additional sensory information, along with the subpatellar bandage. Both groups presented a decrease in results in the TUG test independently of the sensory condition. The statistical tests revealed a significant difference between evaluations with and without additional sensory information (*p* < 0.05) but did not show significance with the use of the subpatellar bandage during video game training.

**Figure 4:**
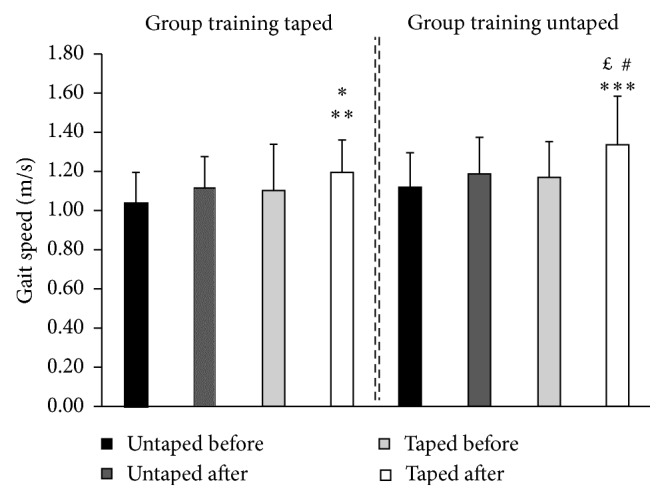
Comparison of gait speed between taped and untaped groups before and after training. Group training taped: ^∗^*p* < 0.001 in relation to untaped before; ^∗∗^*p* < 0.05 in relation to taped before; group training untaped: ^∗∗∗^*p* < 0.001 in relation to untaped before; ^#^*p* < 0.01 in relation to untaped after; ^£^*p* < 0.001 in relation to taped before.

The gait speed data evaluations before and after training with and without the use of the subpatellar bandage are represented in Figure 4. Both groups had an increase in gait speed after training, independently of the sensory condition of the group. The participants also represented an increase in the gait speed during the evaluations after training with the use of additional sensory information.

## 7. Discussion

Research on the benefits brought to the elderly by video game technologies, especially in terms of postural balance and risk of falling, has increased in recent years [28, 29]. Also, it has been shown that the addition of sensory information decreases postural imbalance in young and elderly people [13–19]. There is no evidence that this increasement in sensory input can contribute in a dynamic intervention process, such as in postural balance training with video games, for example. Therefore, the objective of this study was to investigate the effect of balance training with Nintendo Wii technology, with and without the use of additional sensory information (subpatellar bandage), in the functional mobility and gait speed of elderly female fallers.

The results obtained revealed an increase in the gait speed and a decrease in the TUG time in both groups, independently of the sensory condition used. In addition, the effects of the additional sensory information were demonstrated after training in both groups.

The use of the video game technologies in elderly populations presents beneficial results regarding the decrease in risks of falls, fragilities, dependence, and deaths, associated with the aspect of motivation and entertainment, considering it an effective strategy to improve the physical health of the elderly [28, 31].

A study by Fu et al. [32], showed that training with the Nintendo Wii promoted an improvement in reaction timing, quadriceps strength, and body oscillation. These findings were found in the comparison with conventional physiotherapy and virtual reality as a means of potential therapy.

The games give feedback in real time for the participants, which is necessary for learning new abilities, especially for rehabilitation. The visual feedback in real time improves training, when compared to the conventional training, assisting the individual with body movement corrections and alignment [33].

There was a reduction in TUG time after training in both groups. This result shows the efficiency of training proposed in the functional mobility of the elderly who fall frequently. These results corroborate with those of Walker et al. [34], which demonstrated an improvement in the TUG test after training with visual feedback.

Gait speed also improved with the training in both groups. There are few studies associating walking gait speed and the use of video games technologies, but the present study suggests the beneficial effect of this training modality to improve the mean gait speed, especially in the elderly. The gait speed is a kinematical parameter; its assessment is simple, cheap, and reliable and may be interesting to assess the mobility of subjects, being possible to avoid complications such as falling, hospital visits, and possibly death. The research by Hardy et al. [35] and Hollman et al. [36], suggests that the reduction of 0.1 m/s in gait speed could increase the risk of the elderly falling by 7% and that the improvement of mean speed could decrease by 17.7% the risk of deaths.

Although the research demonstrated the efficiency of training with Nintendo Wii, the hypothesis that the additional sensory information could maximize this effect was rejected despite a trend of improvement of the parameters. In contrast, the evaluations demonstrated that the subpatellar bandage presents a good effect in short term.

Only one study evaluated the effects of a sensorimotor training with the use of the additional sensory information showing good results [20]. In our study, this hypothesis has not been verified. One reason for this may be that the training in our study was different from the Botelhos study. Although it cannot be generalized, the results suggest that the use of subpatellar bandage improves balance and mobility and can be used to reduce the risk of falls among the elderly, in a short time. In addition, other studies should be done with a larger number of participants to show whether subpatellar bandage may potentiate the effects of treatments. The video game technologies represent a good acceptance for the geriatric population due to a playful component and the audio-visual feedback given in each game. The facility of using the tool in addition to its mobility improvements could turn video game technologies' training into an important ally to the conventional physiotherapy methods [37–39].

## 8. Conclusion

The training for postural balance with Nintendo Wii balance board, with and without the inclusion of additional sensory information was effective for the improvement of the functional mobility and gait speed of elderly fallers. The subpatellar bandage had a short-term effect, but it was not possible to show its potential to amplify the training effect; however, there was a trend for improvement.

## Figures and Tables

**Table 1:** Sample characterization.

	Group taped (*n*=10)	Group untaped (*n*=10)
	Mean (SD)	Mean (SD)
Age (years)	70.1 (6.33)	69.5 (8.07)
MMSE	24.9 (2.99)	26.2 (2.74)
Number of falls	2.0 (0.66)	1.2 (0.70)

MMSE: Mini-Mental State Examination; SD: standard deviation.
